# Neutrophils initiate proinflammatory immune responses in early endometriosis lesion development

**DOI:** 10.1172/jci.insight.186133

**Published:** 2025-01-21

**Authors:** Taylor R. Wilson, Kurt R. Peterson, Stephanie A. Morris, Damaris Kuhnell, Susan Kasper, Katherine A. Burns

**Affiliations:** 1 Division of Environmental Genetics and Molecular Toxicology, Department of Environmental and Public Health Sciences, and; 2 Division of Reproductive Endocrinology and Infertility, Department of Obstetrics and Gynecology, University of Cincinnati College of Medicine, Cincinnati, Ohio, USA.

**Keywords:** Immunology, Inflammation, Reproductive biology, Mouse models, Neutrophils

## Abstract

Endometriosis is a chronic gynecological disease that affects 1 in 10 reproductive-aged women. Most studies investigate established disease; however, the initiation and early events in endometriotic lesion development remain poorly understood. Our study used neutrophils from human menstrual effluent from patients with and without endometriosis for immunophenotyping, and it used a mouse model of endometriosis and a mouse endometriosis cell line to determine the role of neutrophils in the initiating events of endometriosis, including attachment and survival of minced endometrial pieces. In menstrual effluent from women with endometriosis, the ratios of aged and proangiogenic neutrophils increased compared with controls, indicating a potentially permissive proinflammatory microenvironment. In our endometriosis mouse model, knocking down neutrophil recruitment with α-CXCR2 into the peritoneum decreased endometrial tissue adhesion — supported by decreased levels of myeloperoxidase and neutrophil elastase in both developing lesions and peritoneal fluid. Fibrinogen was identified as the preferred substrate for endometrial cell adhesion in an in vitro adhesion assay and in developing lesions in vivo. Together, aged and proangiogenic neutrophils and their secretions likely promote attachment and formation of endometriotic lesions by releasing neutrophil extracellular traps and upregulating fibrinogen expression as a provisional matrix to establish attachment and survival in the development of endometriosis lesions.

## Abstract 2

Menstrual effluent from women with endometriosis have increased aged and pro-angiogenic neutrophils. In a mouse model, neutrophils are necessary for attachment and survival of endometriosis lesions.

## Introduction

Endometriosis is a chronic gynecologic disease affecting 1 in 10 reproductive-aged women and people who menstruate (1, 2). Endometriosis is the presence of proliferating endometrial-like tissue outside the uterine cavity and affects the quality of life and reproductive health of millions worldwide (3–5). Individuals suffering from endometriosis experience dysmenorrhea, chronic pain (including dyspareunia, dysuria, and dyschezia), and infertility (3, 6) as well as associated comorbidities: gastrointestinal dysfunctions, autoimmune disorders, and increased ovarian cancer risk (7, 8). Diagnosis is often delayed 7–9 years due to the need for laparoscopic surgical confirmation or due to incorrect diagnoses, contributing to uncertainty of the true prevalence of endometriosis (6). Treatments are mainly palliative with no definitive cure (9). Currently, potential biomarker targets for diagnosis lack specificity, leading to ineffective diagnosis and treatment options (10, 11).

The definitive etiology and pathophysiology of endometriosis remains elusive. While the field acknowledges multiple etiologic theories, retrograde menstruation remains a prevailing hypothesis (12, 13). Greater than 90% of women experience retrograde menstruation; however, ~10% develop endometriosis (3), indicating dysfunction in immune regulation and/or indicating that additional factors are required for development and progression. A primary consensus is that estrogens regulate endometriosis progression; however, initiation of endometriosis lesion formation appears to be driven by hormone-independent immune responses (14). Endometriosis is considered an inflammatory condition (3, 14) with altered immune surveillance as a consequence of endometriosis development from incomplete clearance of viable endometrial tissue. Neutrophil levels in the peritoneal fluid (PF) of women with endometriosis are ~4-fold higher compared with healthy women (15), suggesting an increased inflammatory microenvironment within the peritoneal cavity. Recent studies report that neutrophils may regulate the initiation of endometriosis (14, 16–18).

Important for neutrophil recruitment is CXCR2 and its associated factors that are implicated in the initiation of endometriosis (15, 16, 19–21); thus, they are becoming a pathway of interest for determining how neutrophils promote inflammatory conditions driving endometriosis. Neutrophils are key players in immune surveillance and are highly regulated to prevent tissue damage and chronic inflammation in healthy tissues (22–24). They act as first-responding effector cells that infiltrate into sites of injury (i.e., peritoneal cavity) for endometriosis lesion initiation (14, 16, 18). Different neutrophil subtypes and activation stages exist; for example, individuals with endometriosis have increased expression of angiogenic factors in PF (25–28) and increased incidences of autoimmune disorders (7, 29–31). Thus, subtypes of proinflammatory neutrophils (i.e., increase inflammation) and proangiogenic neutrophils (i.e., promote angiogenesis) are of particular interest for understanding mechanisms promoting endometriosis development (32).

In addition to recruitment, CXCR2 is also critical for neutrophil homeostasis and maturation (33, 34). In several inflammatory disease models, blocking or knocking down CXCR2 decreases neutrophil recruitment, tissue damage, and mortality (33). Increased neutrophils in the PF of women with endometriosis and in vivo mouse studies of inflammatory diseases strongly suggest a role for CXCR2 and its ligands in endometriosis development (15, 19–21). Moreover, in patients with endometriosis, CXCR2 is increased in endometrial biopsies shown by microarray and IHC analyses implicating it in the inflammatory profile of endometriosis (20, 21). Neutrophils additionally mediate an adhesive response allowing ectopic endometrial tissue to become lesions; therefore, knocking down CXCR2 may prevent lesion attachment and survival. CXCR2 inhibitors have potential therapeutic advantages; some have progressed to Phase IIa clinical trials without deleterious or irreversible side effects (35, 36). Hence, CXCR2 inhibition could target key processes in disease initiation and possibly serve to prevent lesion development (36–39).

Together with our studies and those of others, we hypothesized that CXCR2 shifts the inflammatory profile of the microenvironment in the peritoneal cavity during the initiation phases of endometriosis, primarily in the attachment and survival of the viable endometrial tissue that subsequently forms lesions. To test the hypothesis, we used our mouse model of endometriosis, which is a surgical dispersion of minced endometrium (SDME) into the peritoneal cavity of hormonally intact mice to capture the cell types present in retrograde menstruation, increase levels of neutrophils and macrophages as observed in women with endometriosis, and develop lesions distally to the surgical site (14, 40–42). Our model is well suited for studying disease initiation, as lesions develop through different stages, are histologically similar to human lesions (i.e., epithelial glands, stroma, hemosiderin macrophages, and fibrosis), and lesions are viable for months (14, 40–42). To compare mouse and human relevance, we collected human menstrual effluent from day 1 of menses to mimic the timing of the early-initiation stage when menstruated endometrium may attach. For timing to correspond to previous studies (14), we selected 24 hours after endometriosis induction for lesion harvest to determine the primary attachment and survival of endometrial tissue. For translational relevance, we selected the first day of menstruation to potentially mimic our experimental mouse model in comparison with human inflammatory responses. Our data provide evidence that women with endometriosis have increased aged and proangiogenic aged neutrophil subpopulations and that neutrophils are contributing effector cells for adhesion and initiation of lesion formation.

## Results

### Aged neutrophil populations in menstrual effluent from women with endometriosis.

Previous findings using our in vivo SDME model demonstrated neutrophil infiltration peaked after the initiation of endometriosis lesion formation following the injection of minced mouse endometrial pieces into the peritoneal cavity at ~24 hours (14). Therefore, 24 hours was selected for our human studies to establish translational relevance to our SDME model to determine whether neutrophils and/or changes in neutrophil subpopulations were also observed in menstrual effluent of women with endometriosis.

**Figure 1:**
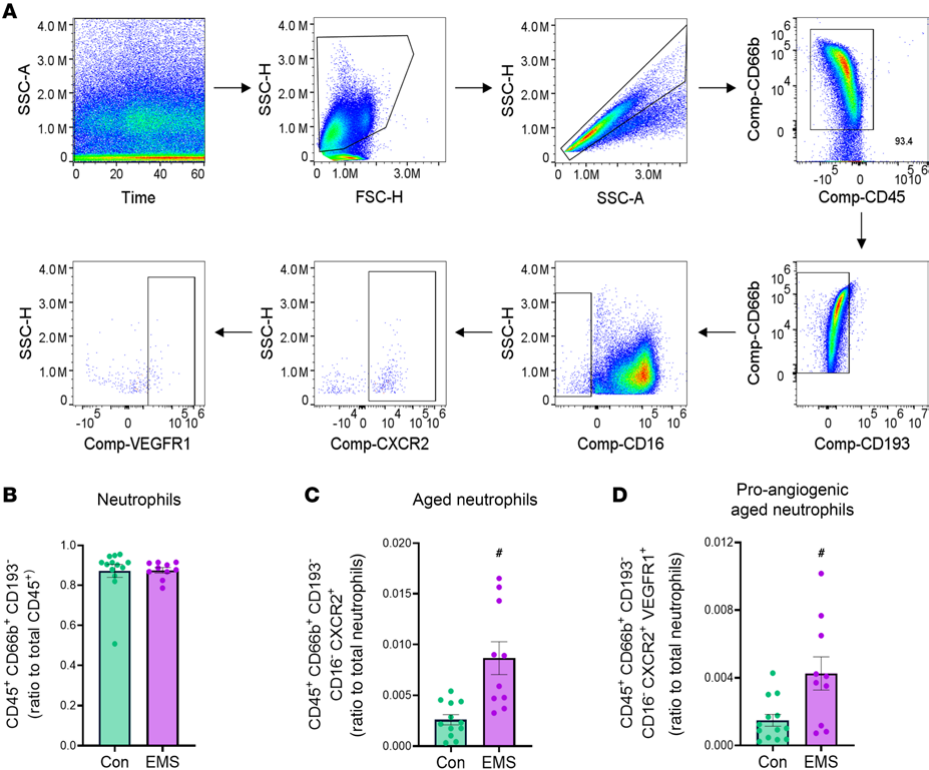
Women with endometriosis have higher levels of aged and proangiogenic neutrophils compared with healthy women. (**A**) Gating strategy of total WBCs isolated from human menstrual effluent. Cells were gated on time, viable cells, single cells, CD45^+^, CD66b^+^, and CD193^–^ to identify total neutrophils. Aged neutrophils were defined as CD45^+^CD66b^+^CD193^–^CD16^–^CXCR2^+^, and proangiogenic neutrophils are defined as CD45^+^CD66b^+^CD193^–^CD16^–^CXCR2^+^VEGFR1^+^. (**B**–**D**) Quantitation of total neutrophils to total WBCs ratio, aged neutrophils to total neutrophil ratio, and proangiogenic aged neutrophils to total neutrophil ratio. Con, healthy controls (*n* = 13); EMS, endometriosis participants (*n* = 10). Data represent ± SEM. Statistical significance for each graph was determined by nonparametric, Mann-Whitney, 1-tailed *U* test. ^#^*P* < 0.05.

Menstrual effluent from women with and without endometriosis was collected on day 1 of menses for immunophenotyping. WBCs were isolated, stained for cellular surface markers, and quantified using gating strategies to isolate granulocytes, exclude eosinophils, and identify potential unique subpopulations of neutrophils associated with endometriosis (Figure 1A). Identified total neutrophils were normalized as a ratio by: calculating total cells per mL and then neutrophils per mL to total leukocytes per mL. No differences in the total neutrophil numbers were observed between endometriosis and control; consequently, neutrophil subtypes were normalized as a ratio by: calculating cells per mL and then aged or aged angiogenic neutrophils per mL to total neutrophils per mL. These ratios accounted for variability in collection volumes and/or time collected per individual. The ratio of neutrophils (CD45^+^CD66b^+^CD193^–^) to total WBCs (CD45^+^) was not different between endometriosis and nonendometriosis control samples (Con) (Figure 1B). In contrast, the ratio of aged neutrophils (CD45^+^CD66b^+^CD193^–^CD16^–^CXCR2^+^) (expressing CXCR2) to total neutrophils (CD45^+^CD66b^+^CD193^–^) increased significantly by 2-fold in endometriosis compared with control (Figure 1C). Moreover, the ratio of proangiogenic neutrophils (CD45^+^CD66b^+^CD193^–^CD16^–^CXCR2^+^VEGFR1^+^) (additionally expressing VEGFR1) increased by ~2.5-fold in endometriosis samples compared with control (Figure 1D). Together, these observations suggest that aged and proangiogenic neutrophils likely play an important role in endometriosis, potentially by providing cytokines and growth factors for the promotion of endometriosis cell survival, adhesion to the peritoneal cavity, and initiation of lesion development, similar to that observed in our mouse model (14). Aged and proangiogenic neutrophils may also participate in the recruitment of additional immune cells (e.g., macrophages, to promote angiogenesis, which would further lesion development) (14).

### Neutrophil knockdown attenuates endometrial attachment to sites in the peritoneal cavity.

**Figure 2:**
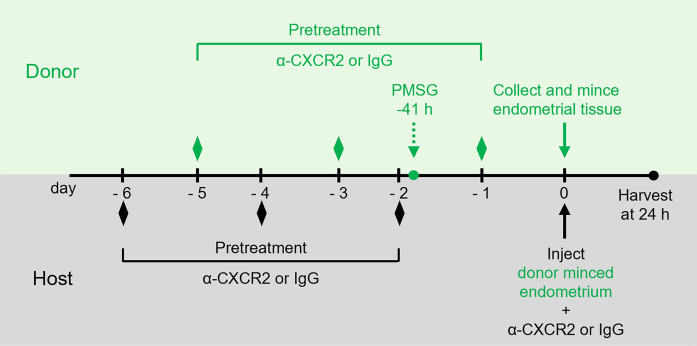
Schematic representation of study treatment and experimental timeline. Donor mice expressing GFP (green) begin treatment bidiurnally with α-CXCR2/IgG 5 days before collection of endometrial tissue (green diamonds). At 41 hours before endometrial collection, donor mice receive an i.p. injection of pregnant mare serum gonadotropin (PMSG) to synchronize uteri. Host mice (black) begin treatment bidiurnally with α-CXCR2/IgG 6 days before surgical induction of endometriosis (black diamonds) with a final dose administered the day of surgery. Mice are euthanized and lesions are collected 24 hours after surgical induction.

**Figure 3:**
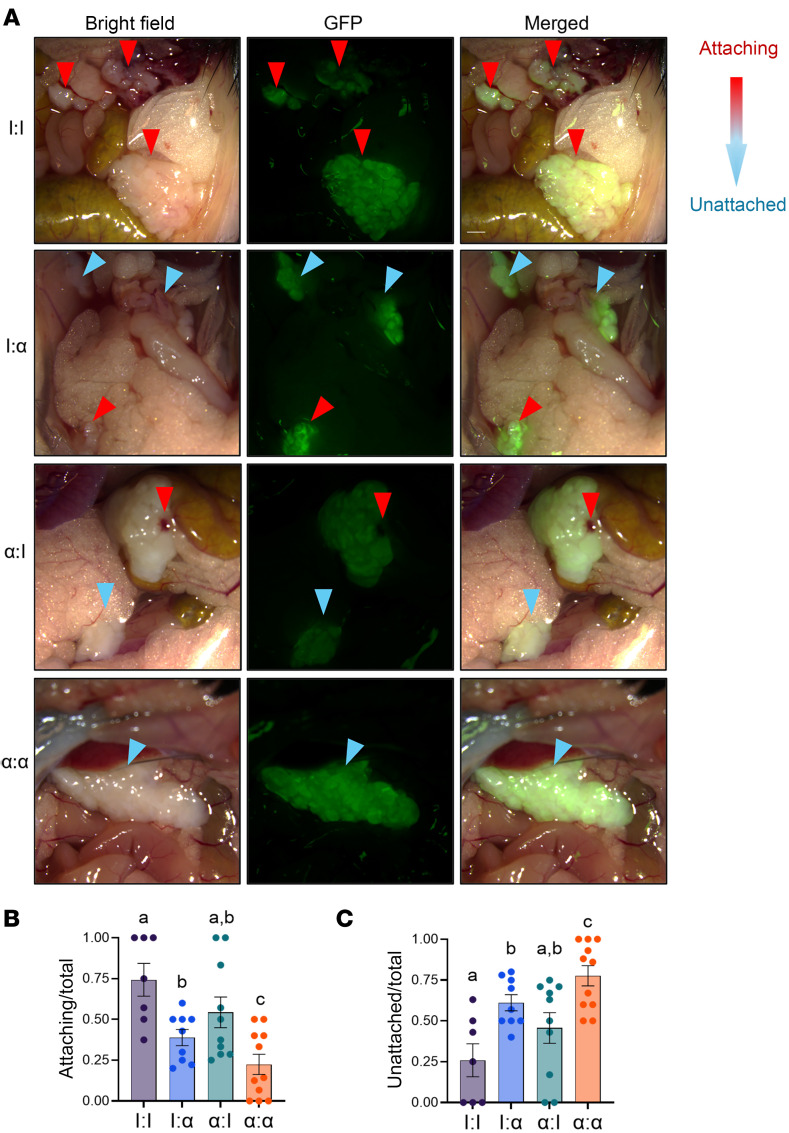
Knockdown of neutrophil recruitment in the SDME model decreased the attachment of minced endometrial pieces at 24 hours to form endometriosis lesions. (**A**) Lesions (24 hours) were derived from GFP minced endometrial pieces and imaged at 488 nm and bright-field from I:I, I:α, α:I, and α:α groups. I, IgG; α, α-CXCR2. Lesions were defined as “attaching” or “unattached” based on color (pink, presence of a blood spot, or attachment for “attaching” versus white and no presence of attachment for “unattached”). Red arrowheads indicate “attaching.” Blue arrowheads indicate “unattached.” Original magnification, 7.5×. (**B** and **C**) Quantitation of attaching and unattached lesions. I:I (*n* = 7), I:α (*n* = 9), α:I (*n* = 10), and α:α (*n* = 11) biological replicates from 2 independent experiments. Data represent ± SEM. Statistical significance for each graph was determined by nonparametric, Kruskal-Wallis followed with 1-tailed Mann-Whitney *U* tests. Letters different from each other are statistically significant, *P* ≤ 0.05.

To determine the effect of neutrophils on endometrial tissue attachment and initiation of endometriosis-like lesion formation (herein called lesions), donor mice expressing GFP were treated with control IgG or α-CXCR2 antibody (2.5 mg/kg in 1× PBS, bidiurnally) 5 days prior to donor endometrial tissue removal to knock down neutrophil recruitment (Figure 2). Minced endometrial tissue pieces from donor mice were dispersed via surgical incision and injection into the peritoneum of GFP^–^ host mice (14, 40–42), and treated with IgG or α-CXCR2 (2.5 mg/kg in 1× PBS, bidiurnally) with pretreatment beginning 6 days prior to endometriosis induction. The donor-to-host (d:h) transfer groups were treated as follows: (a) IgG to IgG (I:I), (b) IgG to α-CXCR2 (I:α), (c) α-CXCR2 to IgG (α:I), and (d) α-CXCR2 to α-CXCR2 (α:α). Twenty-four hours later, developing lesions, PF, and peritoneal cells were collected. At necropsy, using macroscopic analysis, lesions were categorized into 2 groups — i.e., “attaching” or “unattached” — based on coloration (pink/attached or white/unattached), the presence of an external blood spot (early initiation of angiogenesis), and/or the presence of physical attachment to sites in the peritoneal cavity (Figure 3).

In the control I:I group, 74% of lesions were localized to sites of attachment 24 hours after endometriosis initiation, and endometrial tissue was pink, more firmly attached, and often found with the presence of a blood spot and/or a visual external blood supply, indicating that attachment to peritoneal surfaces and lesion formation had been initiated (Figure 3, A and B). Unattached endometrial tissue (26%) was white in color, found floating in the peritoneal cavity or lying superficially on top of peritoneal sites, and no evidence of physical attachment or formation of a visual external blood supply was observed at 24 hours (Figure 3C). To normalize the number of observed lesions per mouse, a ratio was calculated comparing the number of attached or unattached lesions with the total number of lesions per mouse. In the I:α- and α:I-treated groups, 39% and 54% of endometrial tissue, respectively, was found attaching, and attachment decreased further to 22% in the α:α group when compared with the I:I group (Figure 3B). As expected, the unattached (Figure 3C) to attached lesion ratios were inversely proportional based on treatment.

Together, these observations imply that knocking down neutrophil recruitment in host mice (I:α and α:α) decreased the initiation of lesion formation to a greater degree than host mice treated with control IgG (I:I and α:I), demonstrating that neutrophil recruitment was required for minced endometrial pieces to attach and initiate lesion formation. Treatment of donor mice with α-CXCR2 (α:I) decreased lesion attachment by 27% compared with I:I, suggesting a role for donor uterine neutrophils in attachment where donor endometrial tissue recruits neutrophils lesser than the host (i.e., peritoneum). Treatment of host and donor mice with α-CXCR2 antibody (α:α) further decreased (43%) the ability of endometrial tissue to attach to peritoneum compared with I:α, indicating that neutrophils from both host and donor participate in lesion development. Together, donor endometrial tissue plays a smaller role in the recruitment of neutrophils, accounting for the differences between host groups in attaching lesions (27% and 59%, I:I to α:I and α:I to α: α, respectively).

In addition to the use of α-CXCR2 antibody to knock down neutrophil recruitment, we also investigated neutrophil recruitment in *Cxcr2*-KO mice (B6.129S2[C]-*Cxcr2^tm1Mwm^*/J; gifted by Alex Lentsch, University of Cincinnati) and used a selective CXCR2 inhibitor, SB225002 (data not shown). Unfortunately, *Cxcr2*-KO mice exhibit failure to thrive when neutrophil recruitment was knocked out and/or there were compensation phenotypes for neutrophil recruitment (43); importantly, surgical procedures were not well tolerated. In addition, neutrophil recruitment was inconsistently reduced in WT mice treated with SB225002. Thus, due to these factors seen in the *Cxcr2*-KO mouse and SB225002 treatments, all further studies were conducted by acutely knocking down neutrophil recruitment with α-CXCR2 antibody.

### α-CXCR2 treatment reduces neutrophil recruitment into PF and endometriosis lesions.

**Figure 4:**
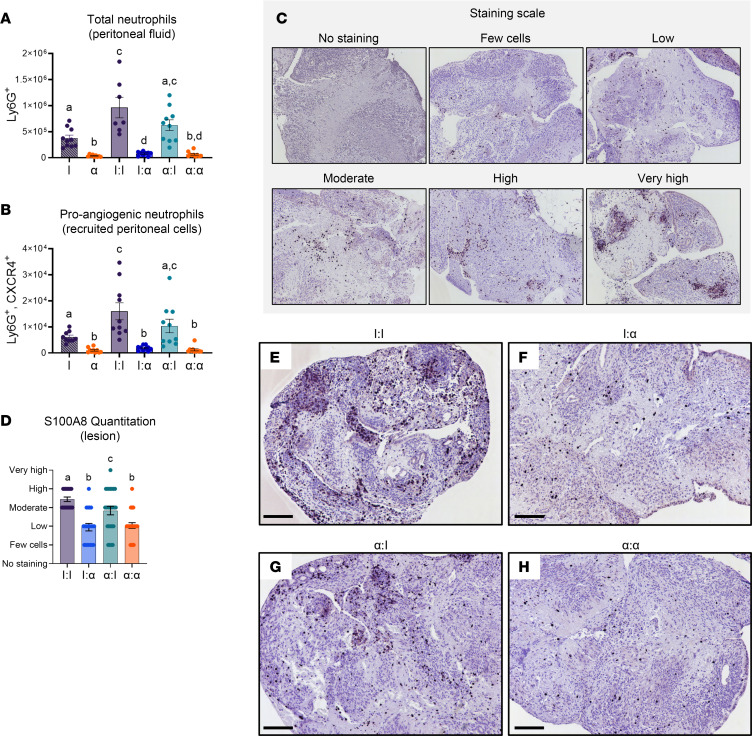
Knockdown of neutrophil recruitment in the SDME model decreased total and proangiogenic neutrophils in peritoneal fluid and lesions. (**A**) Total neutrophils were gated as live cells, single cells, and Ly6G^+^ from I sham (*n* = 10), α sham (*n* = 9), I:I (*n* = 7), I:α (*n* = 9), α:I (*n* = 10), and α:α (*n* = 10) groups from biological replicates from 2 independent experiments. I, IgG; α, α-CXCR2. (**B**) Proangiogenic neutrophils were gated with CXCR4^+^ from the Ly6G^+^ population from I sham (*n* = 9), α sham (*n* = 10), I:I (*n* = 10), I:α (*n* = 10), α:I (*n* = 10), and α:α (*n* = 10) groups from biological replicates from 2 independent experiments. (**C**) Representative images of S100A8 staining scale. (**D**) Quantitation of S100A8 staining in lesions. I:I (*n* = 18), I:α (*n* = 20), α:I (*n* = 25), and α:α (*n* = 24) groups from biological replicates from 2 independent experiments from biological replicates from 2 independent experiments. (**E**–**H**) Lesions stained with S100A8. Dark purple represents neutrophil infiltration into the lesion. Original magnification, 100×. Representative images from I:I, I:α, α:I, and α:α. Data for each graph represent ± SEM. Statistical significance for each graph was determined by nonparametric, Kruskal-Wallis followed with 1-tailed Mann-Whitney *U* tests. Letters different from each other are statistically significant. *P* ≤ 0.05. Scale bar: 100 μm. Original magnification, 100x (**C**, **E**–**H**).

Peritoneal lavage obtained from the I:I, α:I, I:α, and α:α treatment groups were analyzed to determine changes in the levels of neutrophils recruited into the peritoneal cavity. In addition, peritoneal lavage obtained from sham surgical mice — i.e., mice having surgery and only given PBS and pretreated with IgG (“I”) or α-CXCR2 (“α”) — served as baseline inflammatory surgical controls. Neutrophils were immunophenotyped as live population, single cells, and Ly6G^+^ (Figure 4A).

Analysis of neutrophils from the sham surgical I and α groups showed treatment with α-CXCR2 alone significantly decreased recruitment of neutrophils into PF by 10-fold compared with I sham treatment. The addition of endometrial tissue pieces to initiate endometriosis lesion formation increased neutrophil recruitment in control I:I PF 2.5-fold compared with I sham surgical controls. In contrast, I:α and α:α PF showed a 12-fold decrease in neutrophils compared with I:I (Figure 4A), while a modest 1.5-fold decrease in neutrophil recruitment was observed in α:I compared with I:I PF, likely due to the donor endometrial tissue response similarly seen in Figure 3. Together, these findings show that the addition of endometrial tissue pieces is required to promote recruitment of neutrophils into PF, and α-CXCR2 treatment of host mice is sufficient to knock down peritoneal neutrophil levels almost to α sham levels, irrespective of the treatment given to donor animals (e.g., I:α = α:α).

Since external blood spots and early initiation of angiogenesis were observed during tissue attachment and initial lesion formation in I:I mice, we also quantitated levels of proangiogenic neutrophil recruitment into PF. Murine neutrophils expressing CXCR4 in addition to Ly6G are categorized as proangiogenic (32, 44, 45). Analysis of neutrophils (Ly6G^+^CXCR4^+^) determined the pattern of proangiogenic neutrophil recruitment similarly mirrored that observed with total neutrophils (Figure 4B), indicating again that presence of minced endometrial pieces was sufficient to increase peritoneal proangiogenic neutrophil numbers with slight differences between host treatment groups (I:I to α:I and I:α to α:α) due to donor tissue interactions and α-CXCR2 treatment of host mice knocked down proangiogenic neutrophil recruitment in PF.

Next, lesion tissue sections were stained with S100A8 to determine whether neutrophils recruited to the peritoneum had infiltrated into the developing endometriosis lesions. Histopathological staining of lesions was characterized based on a staining scale ranging from no staining to very high staining (Figure 4C). Scores for each lesion were quantitated using the staining scale (Figure 4D). Analysis of lesions from all treatment groups exhibited similar histological structure with a mixture of stromal, epithelial cells, and glandular structures 24 hours after endometrial tissue injection (Figure 4, E–H). Moderate to high numbers of neutrophils were observed in I:I control lesions (Figure 4E), while neutrophil recruitment into I:α (Figure 4F) and α:α (Figure 4H) lesions decreased to low numbers, corresponding to the decreases observed in lesion attachment and peritoneal neutrophil recruitment (Figures 3, A and B, and Figure 4, A and B). In addition, neutrophil recruitment into α:I lesions decreased significantly compared with I:I but significantly increased compared with I:α and α:α, suggesting that donor neutrophils, present in minced endometrial tissue, are contributing effector cells in recruitment of host-derived neutrophils.

In summary, the flow cytometry data indicate that neutrophil recruitment was not fully blocked by α-CXCR2 treatment. Not surprisingly, as neutrophils rapidly differentiate and extravasate from the vasculature during inflammation. This increase in neutrophil recruitment into I:I PF, followed by a significant increase of neutrophils into I:I lesions, demonstrates that introduction of minced endometrial pieces into the peritoneum elicits a physiological immune reaction. Moreover, α-CXCR2 treatment knocks down neutrophil recruitment into PF and lesions, correlating with the decrease in lesion attachment following treatment (Figure 3). Concomitantly, the considerable decrease in proangiogenic neutrophils also correlates with the loss of external blood spots or signs of early initiation of angiogenesis (Figure 3). Taken together, these findings propose that neutrophil recruitment is an important factor in tissue survival, attachment, angiogenesis, and initiation of the development of endometriosis lesions.

### Neutrophil-associated gene expression in endometriosis lesions decreased with α-CXCR2 treatment.

To determine the effect of α-CXCR2 treatment on neutrophils recruited into lesion tissue, we evaluated neutrophil-associated gene expression in I:I, α:I, I:α, and α:α lesions compared with control minced endometrial pieces obtained from IgG-treated (“I”) and α-CXCR2–treated (“α”) donor mice. Neutrophil-associated target genes included *Cxcr2*, *S100a8*, *S100a9*, and *Csf3r*. We observed that *Cxcr2* expression was low in IgG and α-CXCR2 minced endometrial pieces. Thus, given these low levels of expression, *Cxcr2* expression in IgG-treated minced endometrial tissue was normalized relative to ribosomal *RpL7* gene expression to enable comparisons between treatment and endometriosis groups. *Cxcr2* expression increased 8-fold and 9-fold in I:I and α:I lesions compared with I:I, respectively, supporting the increased recruitment of neutrophils observed previously (Figure 4) (14). In addition, *Cxcr2* expression in I:α and α:α lesions decreased by > 4.5-fold and 2-fold, respectively, compared with I:I lesions.

**Figure 5:**
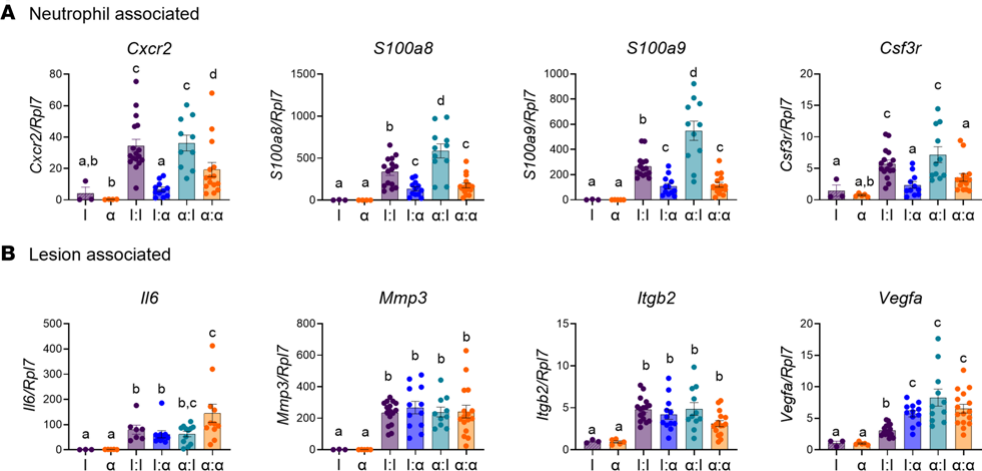
Knockdown of neutrophil recruitment in the SDME model decreased neutrophil infiltration into lesions and decreased further neutrophil recruitment. (**A**) Neutrophil-associated gene expression targets from I uterus, α uterus, I:I, I:α, α:I, and α:α groups. *Cxcr2*: I uterus (*n* = 3), α uterus (*n* = 4), I:I (*n* = 16), I:α (*n* = 11), α:I (*n* = 10), and α:α (*n* = 15). *S100a8:* I uterus (*n* = 3), α uterus (*n* = 4), I:I (*n* = 16), I:α (*n* = 12), α:I (*n* = 11), and α:α (*n* = 16). *S100a9:* I uterus (*n* = 3), α uterus (*n* = 5), I:I (*n* = 15), I:α (*n* = 12), α:I (*n* = 11), and α:α (*n* = 16). *Csf3r:* I uterus (*n* = 3), α uterus (*n* = 5), I:I (*n* = 16), I:α (*n* = 11), α:I (*n* = 11), and α:α (*n* = 15) (**B**) Knockdown of neutrophil recruitment did not alter a subset of genes associated with endometriosis and inflammation, demonstrating uterine-associated processes in lesion development from I uterus, α uterus, I:I, I:α, α:I, and α:α groups. *Il6:* I uterus (*n* = 3), α uterus (*n* = 5), I:I (*n* = 7), I:α (*n* = 10), α:I (*n* = 12), and α:α (*n* = 11). *Mmp3:* I uterus (*n* = 3), α uterus (*n* = 5), I:I (*n* = 16), I:α (*n* = 12), α:I (*n* = 10), and α:α (*n* = 16). *Itgb2:* I uterus (*n* = 3), α uterus (*n* = 5), I:I (*n* = 16), I:α (*n* = 12), α:I (*n* = 11), and α:α (*n* = 16). *Vegfa:* I uterus (*n* = 3), α uterus (*n* = 5), I:I (*n* = 17), I:α (*n* = 12), α:I (*n* = 11), and α:α (*n* = 16). For each graph, biological replicates are shown from 2 independent experiments. Data for each graph represent ± SEM. Statistical significance for each graph was determined by nonparametric Kruskal-Wallis followed with 1-tailed Mann-Whitney *U* tests. Letters different from each other are statistically significant. *P* ≤ 0.05.

S100A8 and S100A9 comprise approximately 45% of the cytoplasmic proteins in neutrophils and are associated with neutrophil recruitment and activation (46–49); CSF3R controls the production, differentiation, and function of granulocytes, including neutrophils (50). Analysis of *S100a8*, *S100a9*, and *Csf3r* gene expression revealed that their expression decreased in parallel with that observed for *Cxcr2* gene expression and that the decrease in I:α and α:α lesions was significantly greater compared with I:I and α:I lesions (Figure 5A). Of note, α:I lesions in *S100a8* and *S100a9* genes showed the overall highest levels of neutrophil-associated target gene expression; although the cause of this was not clear, it may be a result of the host reacting to the α donor tissue. 

In determining the potential role of endometrial-associated gene expression in lesion formation, we initially hypothesized that integrins and selectins would play a major role in the processes of adhesion of endometrial tissue due to their specialized functions in chemotaxis and extravasation during recruitment of neutrophils (51); however, analysis of a select group of selectins (*Sell*, *Selp*) did not show modulation in lesion development in I:I, α:I, I:α, or α:α lesions compared with minced endometrial pieces obtained from I- and α-treated donor mice (data not shown). Interestingly, *Il6*, *Mmp3*, *Itgb2*, and *Vegfa*, a group of genes known to play a role in endometriosis, increased in the developing lesion tissues in all groups compared with control I and α minced endometrial pieces (Figure 5B). Together, these findings indicate that the expression of factors regulating neutrophil activation and function are primarily modulated via neutrophils that have infiltrated into the lesions.

IL-6 is reported as an important cytokine in both the endometrium and endometriosis lesions in women with endometriosis and is known to be both proinflammatory and antiinflammatory (52). *Il6* expression increased by greater than 68-fold in all I:I, α:I, I:α, and α:α lesions compared with I- and α-treated minced endometrial pieces. MMP3 remodels extracellular matrix and is overexpressed in endometriosis, and MMP3 polymorphism increases the risk of developing advanced endometriosis and infertility (53). *Mmp3* expression increased by 200-fold in all I:I, α:I, I:α, and α:α lesions compared with I- and α-treated minced endometrial pieces. Integrin β-2 (ITGB2) is an adhesion molecule known to play a role in regulating neutrophil trafficking and other immunological processes (51). *Itgb2* expression increased by 4-fold in all I:I, α:I, I:α, and α:α lesions compared with I- and α-treated minced endometrial pieces. VEGFA is highest during menstruation in endometriosis compared with normal controls, is elevated in the PF of women with endometriosis, and promotes angiogenesis (54). *Vegfa* expression increased by 3-fold in all I:I, α:I, I:α, and α:α lesions compared with I- and α-treated minced endometrial pieces. Additionally, expression of the integrins *Itga11* increased by 124-fold (data not shown), and this increase was independent of neutrophil knockdown. Factors regulating endometrial tissue survival, adhesion to peritoneal sites, and angiogenesis during initiation of lesion formation are primarily produced by endometrial cells mediated by signals from the endometrium, and these signals can be modulated by neutrophil-dependent and neutrophil-independent mechanisms.

### Vasculature at 24 hours is donor derived.

**Figure 6:**
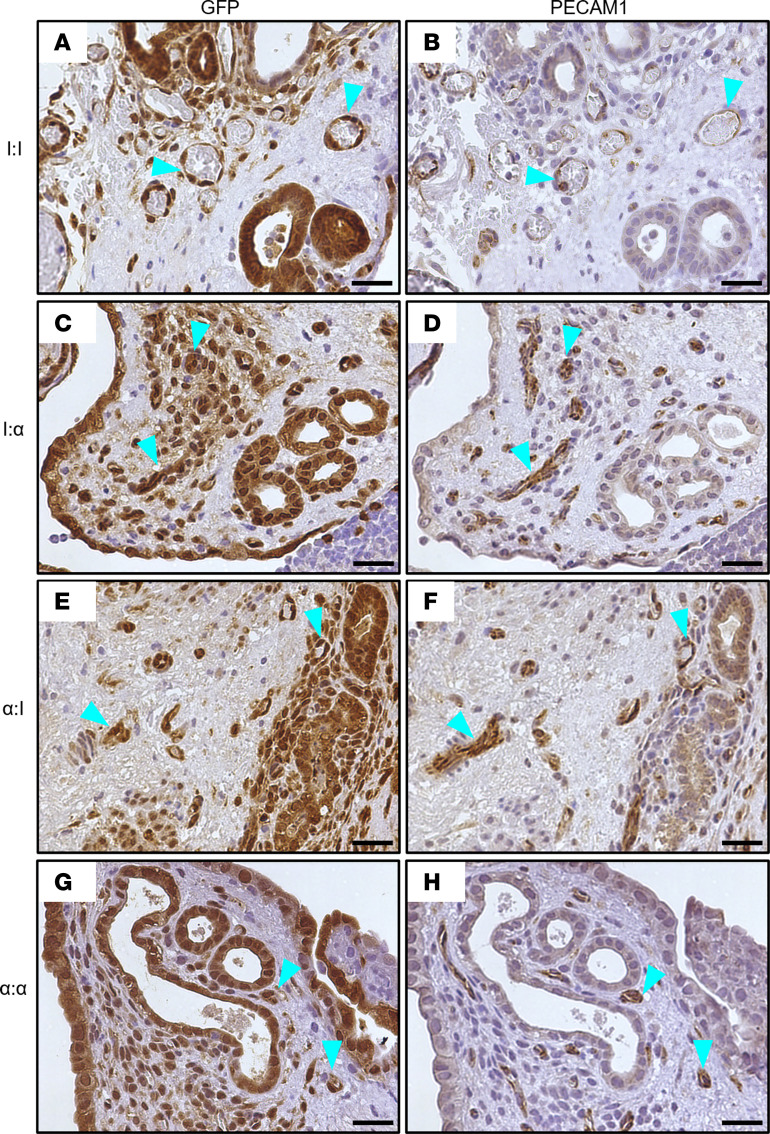
Endometriosis lesions include existing GFP^+^ donor-derived blood vessels and not de novo–derived blood vessels, regardless of neutrophil knockdown 24 hours after endometriosis induction. (**A**, **C**, **E**, and **G**) Developing endometriosis lesions stained for GFP donor-derived tissue are GFP^+^ (brown stain) and contain endometrial glands from minced uterine tissue. (**B**, **D**, **F**, and **H**) Developing endometriosis lesions were stained to visualize endothelial cells lining blood vessels for PECAM1 (brown staining). Representative blood vessels (teal arrowheads) found in developing lesions from serial sections stained for GFP and PECAM1. GFP: I:I (*n* = 12), I:α (*n* = 16), α:I (*n* = 18), and α:α (*n* = 14). PECAM1: I:I (*n* = 12), I:α (*n* = 18), α:I (*n* = 18), and α:α (*n* = 14). Lesions per group represent biological replicates from 2 independent experiments. Scale bar: 25 μm. Original magnification, 400x.

To examine the early initiation of vasculature, lesions from each group were stained with anti-PECAM1 to visualize vasculature and with anti-GFP to validate donor-derived tissue. Histopathological staining of lesions was examined for de novo vasculature since PECAM1 is involved in angiogenesis and is a marker for endothelial junctions. Representative images (Figure 6, B, D, F, and H) from each group are shown. No remarkable differences between the size, location, or formation of vessels at the 24-hour time point was observed. Additionally, staining of lesions with GFP demonstrated that the vasculature in the lesions were donor endometrial tissue derived (Figure 6, A, C, E, and G). This result is comparable with another study that also did not observe de novo angiogenesis in the lesion 72 hours after endometriosis induction (16). Although *Vegfa* (Figure 5B) was elevated in the endometriosis induction groups, the delay between transcription and translation likely caused the delay in visible neoangiogensis within the lesion.

### Neutrophil extracellular traps promote endometrial tissue attachment.

Since knocking-down neutrophil recruitment did not alter adhesion factor expression, neutrophil extracellular traps (NETs) were examined to gain further insight into the mechanisms of lesion attachment. NETs are an inflammatory process resulting in the expulsion of decondensed chromatin, histones, and enzymes to produce a viscous and adhesive meshwork intended to contain an inflammatory incident (55, 56). In response to stress, NETs serve as an adhesive substrate for cells (57) and release the byproduct neutrophil elastase (ELA2), which is essential to initiate NET formation and synergizes with another NET byproduct, myeloperoxidase (MPO), the most abundant proinflammatory biomarker present in neutrophilic granulocytes, to accelerate chromatin decondensation during NET formation (i.e., NETosis) (58, 59). Thus, ELA2 and MPO were evaluated in both PF and crushed lesion tissues via ELISA to determine whether NET formation was a potential mechanism involved in the survival and attachment of endometrial tissue.

**Figure 7:**
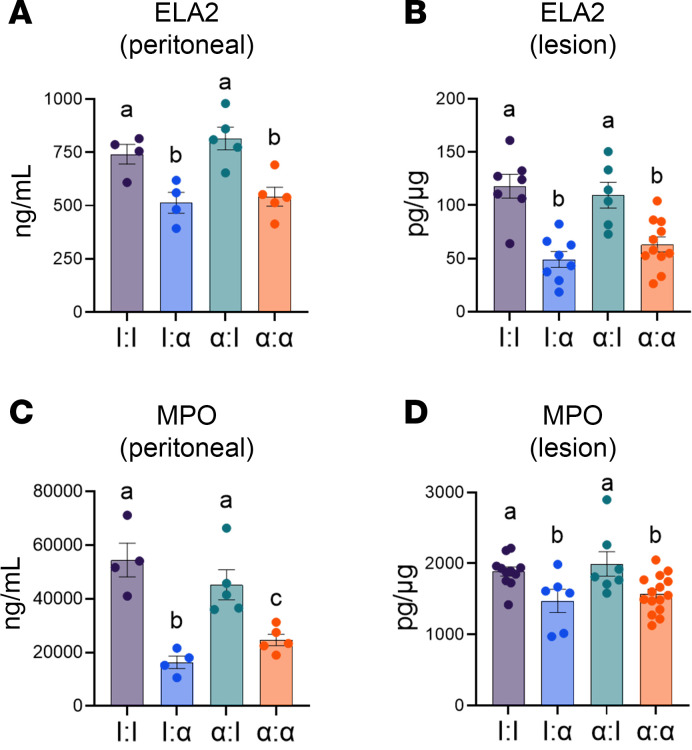
Knockdown of neutrophil recruitment in the SDME model decreased NET formation in peritoneal fluid and developing lesions. (**A**–**D**) ELISA for ELA2 in peritoneal fluid from I:I (*n* = 4), I:α (*n* = 11), α:I (*n* = 5), and α:α (*n* = 5); ELA2 in lesions from I:I (*n* = 7), I:α (*n* = 8), α:I (*n* = 6), and α:α (*n* = 11); MPO in peritoneal fluid from I:I (*n* = 4), I:α (*n* = 4), α:I (*n* = 5), and α:α (*n* = 5); and MPO in lesions from I:I (*n* = 11), I:α (*n* = 6), α:I (*n* = 7), and α:α (*n* = 15). Each graph contains biological replicates from 2 independent experiments. Data for each graph represent ± SEM. Statistical significance for each graph was determined by nonparametric, Kruskal-Wallis followed with 1-tailed Mann-Whitney *U* tests. Letters different from each other are statistically significant. *P* ≤ 0.05.

ELA2 levels decreased by 30% in I:α and by 27% in α:α PF compared with the I:I group, with no changes in α:I compared with I:I (Figure 7A). ELA2 in lesions decreased by 58% in I:α, with no changes in α:I and a 38% decrease in α:α lesions compared with the I:I control group (Figure 7B). MPO decreased by 70% in I:α, with no changes in α:I and a 65% decrease in α:α PF compared with the I:I group (Figure 7C), while MPO decreased by 22% in I:α and by 15% in α:α lesions with no changes in α:I compared with I:I (Figure 7D). Lesions were not compared with eutopic endometrium or sham PF due to reduced neutrophils (i.e., low to no expression of S100A8/A9 in eutopic endometrium). These observations imply that ELA2 likely plays a more prominent role in lesion development, whereas MPO is more important in NET formation in driving the proinflammatory microenvironment provided by the PF.

### Neutrophils initiate extracellular matrix remodeling and adhesion sites for lesions.

**Figure 8:**
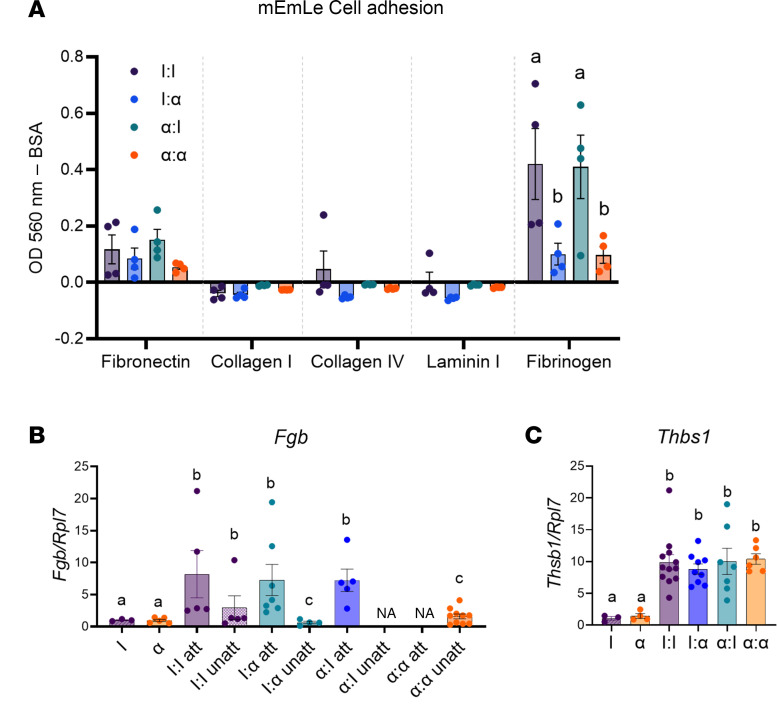
mEmLe cells adhere to fibronectin in a neutrophil dependent manner, and the same fibrinogen response is observed in attaching lesion tissue. (**A**) mEmLe cells were used in an adhesion array with fibronectin, collagen I, collagen IV, laminin I, and fibrinogen. Cells were allowed to adhere in the presence of peritoneal fluid from I:I, I:α, α:I, and α:α groups where I = IgG and α = α-CXCR2. *n* = 4 representative of 3 independent experiments. (**B**) Fibrinogen (*Fgb*) gene expression in minced endometrial pieces, attaching (att), and unattached (unatt) lesions from I uterus, α uterus I:I, I:α, α:I, and α:α groups. I uterus (*n* = 3), α uterus (*n* = 5), I:I att (*n* = 5), I:I unatt (*n* = 5), I:α att (*n* = 7), I:α unatt (*n* = 4), α:I att (*n* = 5), α:I unatt (*n* = NA), α:α att (*n* = NA), and α:α unatt (*n* = 10) for attaching and unattached lesions of biological replicates from 2 independent experiments. (**C**) Thrombospondin (*Thbs1*) gene expression in minced endometrial pieces and lesions from I uterus (*n* = 3), α uterus (*n* = 4), I:I (*n* = 11), I:α (*n* = 9), α:I (*n* = 7), and α:α (*n* = 9) groups. *n* = 3–5 for minced endometrial pieces, *n* = 11–17 for lesions of biological replicates from 2 independent experiments. NA = no gene expression. Data represent ± SEM. Statistical significance for each graph was determined nonparametric, Kruskal-Wallis followed with 1-tailed Mann-Whitney *U* tests. Letters different from each other are statistically significant. *P* ≤ 0.05.

To determine the extracellular matrix (ECM) preferred for lesion adhesion, we used our spontaneously immortalized cell line from mouse endometriosis lesions (mEmLe) (42) in an ECM array assay to delineate lesion attachment at the single-cell level based on their ability to attach to the ECM components fibronectin, collagen I, collagen IV, laminin I, and/or fibrinogen. For this assay, cells were plated in the presence of PF from all groups. Each group was normalized to BSA-coated wells. Fibrinogen is a glycoprotein and component of blood clots that is cleaved by thrombospondin to produce fibrin fibers that provide stability to clots (60). As seen in Figure 8A, mEmLe cells clearly selected fibrinogen as a preferred substrate for adhesion in I:I and α:I PF compared with BSA control. Furthermore, cell attachment decreased by 2.5-fold in both I:α and α:α PF compared with I:I, indicating that decreasing neutrophil recruitment into the peritoneum also decreased attachment at the cellular level. In addition, mEmLe cells attached modestly to fibronectin, a glycoprotein present in plasma that can play a major role in cell adhesion and wound healing (61), in a similar trend seen with fibrinogen. However, mEmLe cells did not attach to collagen I, collagen IV, or laminin over BSA baseline, implying that these ECM components were not essential for lesion adhesion.

Next, we determined changes in the expression levels of the fibrinogen α (*Fga*) and β (*Fgb*) subunits in attached and unattached lesions in response to neutrophil depletion. *Fga* was not expressed in lesions or minced endometrial pieces; however, *Fgb* expression increased > 8-fold in attached I:I, I: α, and α:I lesions compared with I and α minced endometrial pieces, and it decreased in unattached I:I lesions by 2.5-fold compared with I:I attached lesions. *Fgb* expression significantly decreased in unattached lesions and was almost undetectable in unattached I: α and α:α lesions (Figure 8B). Insufficient lesion numbers were unattached in α:I or attached in α:α groups to measure *Fgb* expression. In contrast, expression of *Thbs1*, a glycoprotein involved in hemostasis and cell matrix remodeling (62), was > 10-fold higher in all lesions groups compared with IgG and α-CXCR2 minced endometrial tissue controls (Figure 8C). While other ECM factors may contribute to initiation of lesion attachment, stability, and/or survival at the early 24-hour time point, fibrinogen and, to a lesser extent, fibronectin are most likely key in initiating the lesion attachment process.

## Discussion

**Figure 9:**
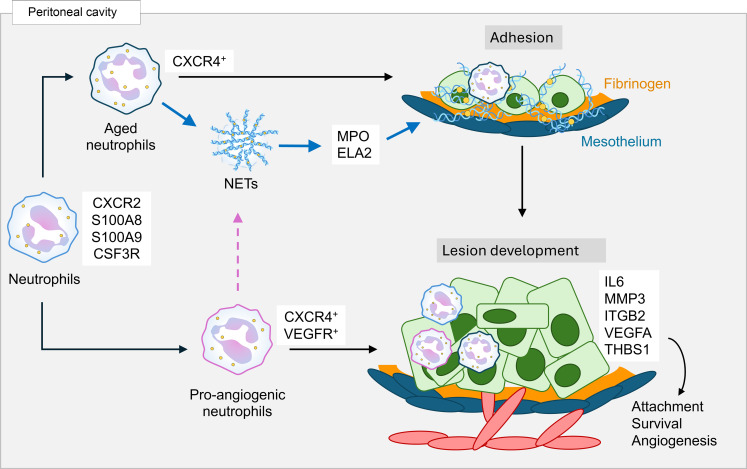
Schematic representation of a proposed mechanism for neutrophil-mediated lesion adhesion and survival. The early development of lesions consists of concurrent neutrophil-dependent and endometrial-dependent phases. Neutrophils recruited to the peritoneal cavity are polarized or activated toward an aged or proangiogenic phenotype (black arrows). Aged neutrophils release NETs (and their byproducts: MPO, ELA2), which respond to fibrinogen to initiate adhesion of minced endometrial pieces and subsequent survival to develop lesions. Proangiogenic neutrophils that may produce NETs and drive survival of minced endometrial pieces through angiogenesis are shown by the dotted pink line. Minced endometrial pieces express survival and adhesive factors to further establish lesion development through attachment, survival, and angiogenesis.

Mechanisms that regulate the early events of endometriosis lesion formation are still poorly understood. We and others have shown that neutrophils and their associated factors are likely effector cells in endometriosis pathogenesis (15, 16, 63, 64). Herein, our study provides evidence that women with endometriosis have an increased ratio of aged and proangiogenic aged neutrophil subpopulations and that neutrophils are required for adhesion and initiation of lesion formation (Figure 9). In addition, NET formation and fibrinogen provide a preferred substrate for the adhesion of endometrial cells that then begin to form endometriosis lesions via the recruitment and the infiltration of neutrophils, the expression of endometrial- and neutrophil-associated factors that promote attachment and survival, and the early external initiation of angiogenesis. Furthermore, the murine study provides evidence of a donor (endometrial) to host (peritoneal) response, supporting the hypothesis that immune cells in the uterus are primed and may alter the immune response in the peritoneal cavity as supported by retrograde menstruation. Importantly, impaired neutrophil recruitment into the peritoneal cavity decreases NET formation, the attachment of endometrial pieces to peritoneal sites, and the initiation of lesion formation in our in vivo SDME model. Identifying the mechanisms that regulate the early stages of endometriosis and discovering biomarkers or targets for preventing lesion attachment and formation would have major effects on the clinical management of endometriosis, since endometriosis disease already begins to clinically manifest itself in young women beginning their periods and current treatment relies on surgical and chemical approaches which are not curative (6).

Traditionally, endometriosis has been considered a hormonally driven disease (40, 65–67). However, our current study implies that the initiation and early stages of lesion development are immune mediated, and we reported previously that introducing endometrial tissue pieces into the peritoneum of immune-compromised mice using our SDME model did not form endometriosis lesions, supporting the concept that a functioning immune system is essential for lesion development (14, 42). Introduction of minced endometrial tissue pieces into the mouse peritoneum elicited a strong immune response, with neutrophils being the initial recruited effector cell into the peritoneum (Figure 4).

Based on our previous study using the SDME model, neutrophil levels peaked 24 hours after induction of endometriosis while macrophages were largely recruited after 48 hours (14); therefore, our current study focused on 24 hours after induction of endometriosis due to the increase and major presence of neutrophils during this time point. Neutrophils are a predominant immune cell type found in menstrual effluent (68); thus, we would predict that retrograde menstruation, which consists of menstrual effluent containing viable uterine tissue, not only provides activated immune cells and their associated cytokines (69, 70) but also triggers the recruitment of neutrophils into the peritoneal cavity to initiate endometriosis formation. Furthermore, the monthly occurrence of menses provides a cycling and/or chronic activation of the immune system, which, in turn, supports the growth of established endometriosis lesions and provides viable endometrial tissue pieces with the opportunity to repeatably establish endometriosis lesions (15, 16, 63, 64). Of considerable interest, retrograde menstruation occurs in nearly all individuals who menstruate; however, only 10% of these individuals develop endometriosis, suggesting that multiple factors, including neutrophils, are at play in endometriosis development (1, 2).

Neutrophils are first responders in a wound-healing environment (71) and participate in uterine homeostatic processes (72). Analysis of neutrophils in menstrual effluent revealed that the ratio of neutrophil/total CD45^+^ immune cells in endometriosis effluent did not differ from control effluent; however, the subpopulation of aged neutrophils in endometriosis effluent increased. Our findings indicate that aged neutrophils are a potentially important maturation state of neutrophils in menstrual effluent from endometriosis women on the first day of menses, implying that these cells are likely a main effector in the initial flow that refluxes into the peritoneal cavity to promote a proinflammatory microenvironment required for establishing endometriosis lesions. Since aged neutrophils are implicated in reactive functions such as NETs and infiltration (73), these neutrophils could attempt to rescue retrograde endometrial tissue in the peritoneum similarly to a wound-healing response, leading to survival and/or reduced clearance of the endometrial tissue.

While macrophages promote angiogenesis in tissue homeostasis and wound repair (74, 75), neutrophils also likely contribute to angiogenesis in endometriosis (63). We have identified an aged neutrophil subpopulation with a proangiogenic subphenotype in endometriosis menstrual effluent that was greater compared with that observed in control effluent. Angiogenesis is essential for providing nutrients, growth factors, and other factors essential for cell survival and to prevent hypoxia (76). Previous studies report that proangiogenic (VEGFR1^+^) neutrophils are recruited to sites of hypoxia (77), and since menstruation is a hypoxic event (72), it is conceivable that the proangiogenic neutrophils in endometriosis retrograde effluent contribute to neoangiogenesis, endometrial cell survival, and ultimately lesion development.

Neutrophils not only clear debris via phagocytosis, but they also recruit additional inflammatory immune cells and induce volatile NETs (55, 56, 78, 79). During inflammation, aged neutrophils readily release NETs (80), which further increase inflammation via the release of NET byproducts, including ELA2 and MPO. In our SDME model, MPO levels were 10-fold higher in lesions and 70-fold higher in PF as compared with ELA2, suggesting that MPO may preferentially enhance the proinflammatory environment in the peritoneal cavity or alternatively, not be cleared as effectively by the immune system. In addition, the findings of NETosis byproducts in early developing lesions suggests that the lack of clearance of the endometrial lesion tissue in women with endometriosis is not only due to the presence of neutrophils in the peritoneal cavity but also to the infiltration of neutrophils into early lesion tissue. NETs may provide an adhesive and permissive microenvironment initiating adherence of endometrial tissue pieces to one another and to peritoneal attachment sites. Thus, therapies that decrease neutrophil recruitment would likely decrease NETosis in both lesions and PF, leading to decreased lesion attachment and, ultimately, decreased survival.

Analysis of factors expressed in developing lesions indicates concurrent neutrophil- and lesion-associated factors independent of each other. Surprisingly, endometrial cells, regardless of α-CXCR2 treatment, expressed *Il6* in the peritoneum, which suggests that the endometrial cells are responsible for recruitment of neutrophils and may encourage proliferation via *Il6* independently from neutrophils. *Mmp3*, *Vegfa*, and *Itgb2* were also increased in developing lesions compared with minced endometrial pieces. *Vegfa* is produced by uterine tissue and macrophages, but our findings suggest that the early initiation of angiogenesis is from the minced endometrial pieces. While in the mouse studies, the transcript for *Vegfa* increased, staining for PECAM1 did not show neoangiogenesis, suggesting that initial lesion vasculature is likely derived from existing donor (GFP) endometrial tissue vessels. In a different mouse model of endometriosis, neoangiogenesis was also not observed during the first 72 hours (16). Interestingly, in attaching lesions, the presence of small external blood spots was observed during necropsy, indicating that neoangiogenesis may begin superficially or externally prior to internal neovascularization (i.e., when uterine tissue is fully attached to the peritoneal site). *Itgb2* was also expected to play a neutrophil-mediated role in lesion development due to cell-surface adhesions properties, but our findings support that integrin responses were independent of neutrophils and are likely a response to endometrial tissue pieces adhering to each other and to sites within the peritoneum. The enzyme MMP3, expressed in normal endometrial cells, degrades ECM components, including fibronectin, laminin collagens, proteoglycans, and elastin important in tissue remodeling and wound repair (81), and it is found in high concentration in menstrual fluid (82). Interestingly, we found that *Mmp3* expression dramatically increased in endometriosis lesion tissue and that fibrinogen was the preferred substrate for endometrial cell adhesion. Together, the increase in neutrophils and NET formation in combination with fibrinogen could provide an endometriosis-specific molecular glue for promoting endometrial cell attachment to peritoneal sites.

Considering that endometriosis lesion development may be a wound-healing event, a 3D network is likely essential for hemostasis with the mechanical properties of clots essential to the functions of fibrin (83). Fibrinogen binds to neutrophils, and the proteases released from them is triggered by fibrinogen degradation (83). Fibrinogen is converted to fibrin via thrombin-mediated proteolytic cleavage (60). Clinical observations supporting the importance of fibrin in endometriosis include: increased thrombin and plasminogen activator inhibitor (PIA1) and sera form denser fibrin clots (84) in women with endometriosis. Also, a FGB polymorphism promotes increased risk of endometriosis (85). Since hemostasis begins immediately after an injury with the formation of a platelet plug that is closely followed by a provisional fibrin matrix to help mediate the wound healing process (83), we hypothesize that the provisional matrix that promotes endometriosis lesion adhesion and lesion development is generated via neutrophil migration, proliferation, and NET formation at attachment sites containing fibrinogen to ultimately support endometrial attachment, survival, and angiogenesis in endometriosis. Identifying the mechanisms that regulate these processes could provide urgently needed biomarkers for determining endometriosis in a disease that takes 7–10 years to diagnose (6). In addition, discovery of targets for preventing lesion attachment and formation would provide more effective approaches in clinical management and prevention of endometriosis where current treatments are prophylactic at best.

## Methods

### Sex as a biological variable.

Studies included both humans and animal models. Only females of each species were part of our study design, because endometriosis is a gynecological disease that occurs in people who menstruate (i.e., genetically XX). In line with using human female samples, all mouse experiments were done using female donor and host animals.

### Menstrual effluent collection and processing.

Menstrual effluent was collected for 6–10 hours on day 1 of menses using a DIVA menstrual cup. Day 1 was characterized by active bleeding, not spotting. After collection, samples were refrigerated and transported on ice. Eligible participants were naturally menstruating, not pregnant, were ages 21–44, did not have an intrauterine device or used hormone contraceptives 3 months prior to collection, had no history of cancer, had no active infections, were not currently on antibiotics, and had no known autoimmune disease. Patients with endometriosis were laparoscopically confirmed. Patient demographics were as follows: Healthy (1 Black, 2 Asian, 10 White; age 25–44 years), Endometriosis (10 White, age 21–40 years). Menstrual effluent was filtered through sterile gauze to remove clots before being centrifuged for 30 minutes at 277*g* to separate the acellular and cellular fractions. Cells were resuspended in PBS + 0.5% BSA + 2 mM EDTA buffer, spun, and filtered via 70 μm and then 40 μm filters to remove epithelial/stromal cells and small clots. Immune cells were isolated via density centrifugation composed of equal parts Histopaque (11191, Sigma-Aldrich) and LymphoprepTM (07851/07861, STEMCELL Technologies) for 20 minutes at 277*g*. The leukocytes were removed from the topmost cellular layer, washed, and utilized for immunophenotyping using spectral flow cytometry. Due to variability in menstrual flow and number of hours collected, samples were normalized per mL of fluid collected and the ratio was determined by the number of neutrophils in each subpopulation per mL corresponding to the total number of neutrophils per mL.

### Animal husbandry.

A syngeneic mouse model of endometriosis was used for induction using hormonally intact host mice (41, 42). Host mice (C57BL/6J) were purchased from Jackson Laboratory. Donor mice (C57BL/6-Tg[UBC-GFP]30Scha/J mice [GFP]) were purchased from The Jackson Laboratory and bred in house. Experimental mice were 8- to 16-week old females housed on sani-chip bedding (phytoestrogen-free) in a controlled temperature range (22°C–23°C) on a 12-hour light/dark cycle. Mice were given food (phytoestrogen reduced 5V5R, LabDiet) and water ad libitum.

α*-CXCR2 antibody*. All mice were randomly assigned to treatment groups for each experimental replicate. Host and donor mice were administered an i.p. injection (2.5 mg/kg diluted in PBS) of either monoclonal rat IgG anti–mouse CXCR2/IL-8RB antibody (α-CXCR2 Ab) or rat IgG_2A_ isotype control antibody (MAB2164 or MAB006, R&D Systems). Donor pretreatment began 5 days prior to endometriosis induction, and mice were dosed bidiurnally for a total of 3 doses before uterine tissue removal. Host pretreatment began bidiurnally for 6 days prior to endometriosis induction, ensuring that mice were dosed on the day of endometriosis induction. On the day of induction, treatments were added to minced uterine endometrium for injection into the peritoneal cavity. Groups are denoted as donor:host. Experimental pairings included: I:I (*n* = 8), I:α (*n* = 9), α:I (*n* = 10), and α:α (*n* = 10). Necropsy was conducted 24 ± 1 hours after endometriosis induction.

### Induction of endometriosis and lesion collection.

Donor mice were synchronized with i.p. pregnant mare serum gonadotropin (PMSG; 3.25 IU), and uteri were removed 41 hours after PMSG administration. To obtain endometrium, myometrium with attached blood supply was peeled away, and endometrium was slit longitudinally and minced (1–2 mm pieces in total ~50 mg) in sterile PBS in glass dishes. Concurrently, a host mouse was prepped using isoflurane anesthesia and administered buprenorphine (0.1 mg/kg) for pain management. Uterine tissue was suspended in 500 μL of PBS with antibody and was then disseminated into the peritoneal cavity of the host through a 3–5 mm dorsolateral hole. To avoid loss of transferred tissue into the subfascial/preperitoneal plane, peritoneal cavity was pulled closed and held with surgical clamps for 30 seconds before the skin was closed with a 9 mm surgical clip. An equivalent amount of minced endometrial tissue was transferred in to all recipients. Donor uterine tissue was transferred at a 1:1 ratio; however, to ensure normalization of tissue amount across groups, 1 uterine horn from 2 different donor mice were combined to meet that 1:1 ratio. All animals were randomized and blinded to the surgeons during surgery and necropsy.

Mice were surgically timed and euthanized 24 ± 1 hours after endometriosis induction. At necropsy, peritoneal lavage was performed to collect peritoneal cells and PF by injecting 1 mL of PBS + 0.5% BSA + 2 mM EDTA (PF buffer) into the peritoneal cavity. The cavity was gently massaged (22 times), the mouse was partially degloved, a small incision was made into the peritoneal cavity, and the fluid was gently removed. After a 150 μL aliquot was removed for differentials, the PF was immediately spun at 1,200 rpm for 5 minutes at 4°C and the supernatant was flash frozen on dry ice and stored at –80°C until use; the cell pellet was resuspended in PBS + 0.5% BSA + 2 mM EDTA and kept on ice until antibody staining. To assess the α-CXCR2–targeted treatment effect on ectopic endometrial tissue development into endometriotic lesions, ectopic tissues were photographed to document in situ images of endometriotic tissue lesions (M250FA with DFC450 camera, LAS X v.3.7.4, Leica Microsystem). Endometriotic lesions were visualized, dissected, measured, and weighed. Tissue was removed with surrounding tissue for histology or without surrounding tissue for gene expression analysis by real-time PCR and either fixed in 10% formalin or flash frozen on dry ice and stored at –80°C until use, respectively. Metrics to compare treatment effects on lesions include lesion number, lesion location, lesion size/weight, lesion volume, and lesion color.

### Flow cytometry analysis.

For mouse experiments, immune cells were isolated from the peritoneal cavity via peritoneal lavage. PF cells were spun at 1,200 rpm for 5 minutes at 4°C and resuspended in 1 mL of FACS buffer (0.5% BSA + 0.1% sodium azide [NaN_3_] + 2 mM EDTA in PBS). The cells were counted by hemacytometer; 2 million cells from each host mouse were plated in 96-well round bottom plates. For single-color and no-staining controls, a mixture of cells from all groups were plated 1 million cells/well. Cells were spun (300*g* for 5 minutes at 4°C), decanted, and suspended in block for 30 minutes in a nonspecific blocking reagent in FACS buffer with 5% normal mouse serum (015-000-120; Jackson ImmunoResearch), 5% normal rat serum (012-000-120; Jackson ImmunoResearch), and 5 mg/mL anti-CD16/32 (2.4G2 hybridoma, BD Biosciences) (86). Antibodies in FACS buffer were added to the samples, mixed, and incubated for 30 minutes on ice (Table 1). Stained cells were analyzed on a LSR Fortessa (BD Biosciences). Neutrophils were immunophenotyped as follows: proinflammatory (Ly6G^+^) and proangiogenic (Ly6G^+^CXCR4^+^). F4/80 and CD11b were used as a control to ensure macrophages were not included. Data were analyzed using FlowJo software (Tree Star Inc.).

For human studies, immune cells isolated from menstrual effluent were isolated and resuspended in with PBS + 0.5% BSA + 2 mM EDTA. In each well, 2 million cells were plated, blocked with 0.5% normal rat serum + 0.5% normal mouse serum + 0.004% anti–human Fc receptor binding (14-9161-73, Invitrogen) in PBS + 0.5% BSA + 2 mM EDTA for 30 minutes, and then stained for cell surface markers identifying neutrophils. CXCR2 and CD193 were first added, one at a time for 10 minutes each, before adding the additional cell surface markers as a mixture for 30 minutes (Table 2). After antibody staining, cells were fixed with 1% paraformaldehyde for 5 minutes. Aged neutrophils were defined as CD45^+^CD66b^+^CD193^–^CD16^–^CXCR2^+^, and proangiogenic aged neutrophils were defined as CD45^+^CD66b^+^CD193^–^CD16^–^CXCR2^+^VEGFR1^+^. Stained cells were analyzed using spectral flow cytometer on the FACS Aurora (BD Biosciences). Single-stained controls for each cell surface marker were used to validate antibodies, unmixing, and compensation using FlowJo software for analyses.

### IHC analysis.

Formalin fixed developing endometrial lesion tissues were routinely processed for paraffin embedding. Sections (3 μm) were cut, and slides were used for IHC. Slides were deparaffinized and hydrated through descending grades of alcohol followed by rehydration to dH_2_0. Sections were refixed with 10% formalin for 10 minutes before heat-induced epitope retrieval. Antigen retrieval was performed using 1× DIVA Decloaker (Biocare Medical) at 95°C for 30 minutes in a decloaking chamber, allowed to cool to room temperature for 10 minutes, and rinsed with dH_2_0. For washes between steps, PBS or PBS with 0.01% Tween-20 (PBS-Tw) was utilized as noted. Avidin-biotin blocking kit (SP-2001, Vector Laboratories, RRID:AB_2336231) was performed following manufacturer instructions. Protein blocking consisted of 5% normal goat serum in PBS-Tw for 1 hour at room temperature. IHC staining for antibodies (Table 3), diluted in Van Gogh Yellow diluent (PD902L, Biocare Medical), was performed for 1 hour at room temperature, followed by PBS-Tw washes. Rabbit normal immunoglobulin (Table 3) was used as a negative control. Secondary antibody incubation was performed with Biotin-SP-AffiniPure F(ab’)2 Fragment goat anti–rabbit IgG (Table 3; 1:200; 111-066-144, Jackson ImmunoResearch, RRID:AB_2337970) in Van Gogh Yellow for 30 minutes followed by PBS-Tw washes and a final PBS wash. Endogenous peroxidase was blocked by incubating slides with 3% hydrogen peroxide for 30 minutes, followed by PBS washes. Tertiary conjugation of ExtraAvidin-Peroxidase (1:100; e2886, Sigma-Aldrich, RRID:AB_2620165) was incubated for 6 minutes, followed by PBS-Tw washes and a final wash with PBS. ImmPact VIP Substrate Kit (SK-4605, Vector Laboratories, RRID:AB_2336525) was used to visualize S100A8 localization. ImmPACT DAB EqV Substrate Kit (SK-4103, Vector Laboratories, RRID:AB_2336521) was used to visualize GFP and PECAM1 localization. All slides were counterstained in Hematoxylin QS (H-3404, Vector Laboratories, RRID:AB_2336843), 1:1 in dH_2_0 for 30 seconds, blued in tap water, dehydrated, and coverslipped.

Sections were imaged at 100× using a Nikon TE300 microscope and DFC450c camera with LAS X v.3.7.4 software. Neutrophil quantification was performed similarly to Jones et al. (41). Each lesion was scored blindly for positive anti-S100A8 neutrophils as follows: no staining, few cells, low, moderate, high, very high. Scores were then averaged and combined.

### RNA isolation and real-time PCR.

Frozen endometriotic tissue lesions from mice were pulverized on dry ice, and RNA was isolated using TRIzol per manufacturer’s instructions (Invitrogen). Lesions containing both attached and unattached sections were excluded from the *Fgb* analysis resulting in a lower *n*. Using a previously described method (42), complementary DNA was synthesized and analyzed by real-time PCR using Fast SYBR. Relative transcript levels were quantified in comparison with endometrium treated with PMSG from respective antibody treatment groups and normalized to *Rpl7*. Primer sequences (Table 4) purchased from Sigma-Aldrich or Thermo Fisher Scientific were selected using Primer Express (Applied Biosystems), Harvard Primer Bank (Harvard University, Cambridge, Massachusetts, USA), PrimerBot! (McDonnell Laboratory, Duke University, Durham, North Carolina, USA), or NIH Primer Blast.

### ELISA.

During necropsy, PF was collected via peritoneal lavage and was centrifuged at 277*g* for 5 minutes to separate acellular and cellular fractions. The flash-frozen acellular fraction was stored at –80°C until analysis. Lesions were cleared of connecting attachments and tissues prior to flash freezing. Representative lesions throughout the peritoneal cavity were selected. The products of NETs tested were ELA2 and MPO. Each ELISA was conducted following manufacturer’s protocol (4517-SE, R&D Systems; Ab285307, Abcam).

### Cell culture.

Cells were cultured in standard conditions at 37°C and 5% CO_2_. The mEmLe cells (derived from GFP expressing mEmLe in the Burns Laboratory) (42) were cultured in DMEM + 10% FBS + 50% conditioned DMEM + 1× penicillin/ streptomycin + fungizone. Standard culture plates were used, and cells were passaged at 90%–100% confluence. The mEmLe cells require a feeder layer derived from peritoneal mesothelial cells (pMeso) developed in the Burns Laboratory. The pMeso cells are host derived, nonfluorescing cells that proliferate more quickly than the GFP fluorescent mEmLe cells; plates frequently underwent microscopic evaluation under 488 nM to remove areas of predominantly nonfluorescent cells.

### Cell attachment assay.

mEmLe cells were serum starved (DMEM + 2.5% sFBS + 1× antibiotic/antimycotic) overnight (18 hours). Cell adhesion/ECM array assay plates were purchased from Cell Biolabs precoated with fibrinogen, collagen type I, fibronectin, collagen type IV, laminin, and BSA. PF from 24-hour experiments (i.e., I:I, I:α, α:I, α:α) was mixed 1:4 following manufacturer’s instructions, cells were resuspended in the medium plus PF mixture, and cells were plated 100,000/well into the ECM array 48-well plates. mEmLe cells were allowed to attach for 1 hour and processed following manufacturers protocol. The wells precoated with BSA served as a negative control and baseline for each treatment group with readings graphed as: experimental OD 560 nM – BSA OD 560 nM.

### Statistics.

When comparing 3 or more groups for multiple comparisons, nonparametric data were analyzed using a Kruskal-Wallis test with the mean rank of each column compared with the mean rank of every other column. If the Kruskal-Wallis test was significant (*P* < 0.05), then Mann-Whitney, 1-tailed *U* tests were performed for validation. Statistical analyses were performed using GraphPad Prism version 10.2.0 (GraphPad Software). When comparing 2 groups, nonparametric, Mann-Whitney, 1-tailed *U* tests were performed. For multiple-group comparisons, means not sharing a letter are significantly different from each other (*P* < 0.05) and means sharing the same single letter or a letter in combination with other letters are not significantly different from each other. For *U* tests, the number symbol (#) indicates significant differences (*P* < 0.05).

### Study approval.

All human studies were approved and conducted in accordance with the University of Cincinnati and Cincinnati Children’s Medical Center IRB (no. 2015-7749). Patients gave written informed consent prior to participation in the study. All animal studies were approved and conducted in accordance with the University of Cincinnati’s IACUC (no. 20-11-24-01).

### Data availability.

Values for all data points associated with the manuscript are provided in the Supporting Data Values file (supplemental material available online with this article; https://doi.org/10.1172/jci.insight.186133DS1). Additional information and detailed methods are available upon request to the corresponding authors.

## Author contributions

TRW, SAM, and KAB performed human menstrual fluid collection, flow cytometry, and data analysis. SAM and KRP performed gene expression analysis and attachment assays. DK performed and analyzed ELISA. SK and KAB developed the mEmLe cell line. TRW, KRP, SAM, DK, and KAB performed surgery and necropsy on mice. TRW, SAM, and KAB independently scored IHC. KAB oversaw the design and analysis of studies performed by TRW, KRP, SAM, and DK. TRW and KAB wrote the paper with comments from KRP, SAM, DK, and SK.

## Supplementary Material

Supporting data values

## Figures and Tables

**Table 1:**
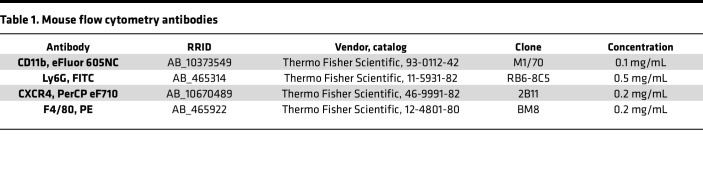
Mouse flow cytometry antibodies

**Table 2:**
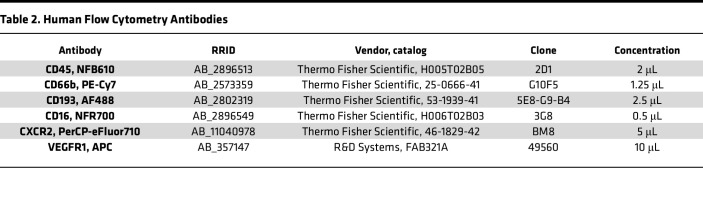
Human Flow Cytometry Antibodies

**Table 3:**
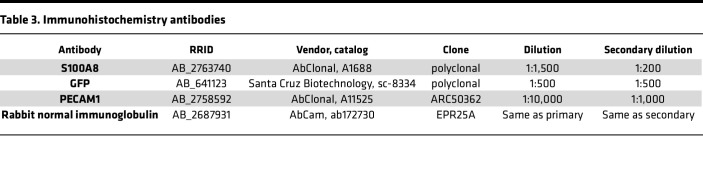
Immunohistochemistry antibodies

**Table 4:**
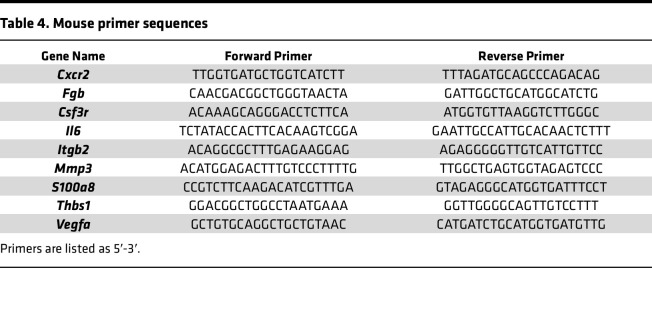
Mouse primer sequences

## Version 1 01/21/2025

In-Press Preview

## Version 2 03/10/2025

Electronic publication
